# Cardiac involvement in Beagle-based canine X-linked muscular dystrophy in Japan (CXMD_J_): electrocardiographic, echocardiographic, and morphologic studies

**DOI:** 10.1186/1471-2261-6-47

**Published:** 2006-12-04

**Authors:** Naoko Yugeta, Nobuyuki Urasawa, Yoko Fujii, Madoka Yoshimura, Katsutoshi Yuasa, Michiko R Wada, Masao Nakura, Yoshiki Shimatsu, Masayuki Tomohiro, Akio Takahashi, Noboru Machida, Yoshito Wakao, Akinori Nakamura, Shin'ichi Takeda

**Affiliations:** 1 Department of Surgery I, School of Veterinary Medicine, Azabu University, Fuchinobe, Sagamihara, Kanagawa, 229-8501, Japan; 2 Department of Molecular Therapy, National Institute of Neuroscience, National Center of Neurology and Psychiatry, 4-1-1 Ogawa-higashi, Kodaira, Tokyo 187-8502, Japan; 3 Chugai Research Institute for Medical Science, Inc., 6598 Toyoda, Suwa, Nagano 392-0016, Japan; 4 Division of Laboratory Animal Resources, National Institute of Neuroscience, National Center of Neurology and Psychiatry, 4-1-1 Ogawa-higashi, Kodaira, Tokyo 187-8502, Japan; 5 Department of Veterinary Pathology, Faculty of Agriculture, Tokyo University of Agriculture and Technology, 3-5-8 Saiwai-cho, Fuchu, Tokyo 183-8509, Japan

## Abstract

**Background:**

Cardiac mortality in Duchenne muscular dystrophy (DMD) has recently become important, because risk of respiratory failure has been reduced due to widespread use of the respirator. The cardiac involvement is characterized by distinctive electrocardiographic abnormalities or dilated cardiomyopathy, but the pathogenesis has remained obscure. In research on DMD, Golden retriever-based muscular dystrophy (GRMD) has attracted much attention as an animal model because it resembles DMD, but GRMD is very difficult to maintain because of their severe phenotypes. We therefore established a line of dogs with Beagle-based canine X-linked muscular dystrophy in Japan (CXMD_J_) and examined the cardiac involvement.

**Methods:**

The cardiac phenotypes of eight CXMD_J _and four normal male dogs 2 to 21 months of age were evaluated using electrocardiography, echocardiography, and histopathological examinations.

**Results:**

Increases in the heart rate and decreases in PQ interval compared to a normal littermate were detected in two littermate CXMD_J _dogs at 15 months of age or older. Distinct deep Q-waves and increase in Q/R ratios in leads II, III, and aVF were detected by 6–7 months of age in all CXMD_J _dogs. In the echocardiogram, one of eight of CXMD_J _dogs showed a hyperechoic lesion in the left ventricular posterior wall at 5 months of age, but the rest had not by 6–7 months of age. The left ventricular function in the echocardiogram indicated no abnormality in all CXMD_J _dogs by 6–7 months of age. Histopathology revealed myocardial fibrosis, especially in the left ventricular posterobasal wall, in three of eight CXMD_J _dogs by 21 months of age.

**Conclusion:**

Cardiac involvement in CXMD_J _dogs is milder and has slower progression than that described in GRMD dogs. The distinct deep Q-waves have been ascribed to myocardial fibrosis in the posterobasal region of the left ventricle, but our data showed that they precede the lesion on echocardiogram and histopathology. These findings imply that studies of CXMD_J _may reveal not only another causative mechanism of the deep Q-waves but also more information on the pathogenesis in the dystrophin-deficient heart.

## Background

Duchenne muscular dystrophy (DMD) is a common and lethal genetic disease characterized by progressive muscle wasting. It is an X-linked recessive disorder caused by mutations in the dystrophin gene, which encodes a cytoskeletal protein, dystrophin [1]. The absence of dystrophin is accompanied by a loss of dystrophin-glycoprotein complex at the sarcolemma and results in progressive degeneration of skeletal and cardiac muscle with fibrotic tissue replacement and fatty infiltration [2,3]. The onset of the disease occurs between 2 and 5 years of age, and most patients die of respiratory or cardiac failure [4,5]. Cardiac involvement, which occurs commonly in DMD patients, has increasingly become an important cause of death because recent clinical progress has reduced the risk of death due to respiratory failure [6,7].

Like dystrophin-deficient skeletal muscle, dystrophin-deficient cardiac muscle is replaced by fibrotic or fatty tissue, especially in the left ventricular posterobasal wall region [8-11]. Atrophic changes with loss of striation, vacuolation, fragmentation, or nuclear degeneration in the myocardium have also been reported [12]. Progressive involvement of the left ventricle leads to wall motion abnormality and results in dilated cardiomyopathy. In DMD patients, the electrocardiogram (ECG) may show tall R-waves in the right precordial leads, deep Q-waves in leads I, aVL, V5-6 or II, III, and aVF [8-13], as well as an increased heart rate, shortened PQ (PR) interval, conduction abnormalities or arrhythmias such as sinus arrhythmia, atrial ectopic beats, and ventricular premature complexes in DMD patients [13-16]. One of the electrocardiographic abnormalities, deep Q-waves, has been considered to be attributable to myocardial fibrosis [8,9,17]. Echocardiography indicates myocardial thickening, wall motion abnormalities, enlargement of the left ventricle, and left ventricular systolic and diastolic dysfunction. Hypokinesis of the posterobasal wall is consistent with the spreading fibrosis and significant decrease in the internal dimensions of the ventricles [14,15]. There are, however, many unresolved issues in cardiac involvement, such as the reason why the posterobasilar segment of the left ventricle is consistently the first lesion, whether extensive fibrosis involves the conduction system, the pathogenesis of inappropriate tachycardia or electrocardiographic abnormalities, and whether abnormal smooth muscle regulation affects the cardiomyopathy [18]. One way to clarify these problems is to study suitable animal models.

To date, the X-linked muscular dystrophy (*mdx*) mouse and the Golden retriever-based muscular dystrophy dog (GRMD) have been used for elucidation of the pathogenesis and development of therapy for DMD. The phenotypes of GRMD are more similar to DMD than that of the *mdx *mouse [19-21], and GRMD also shows similar electrocardiographic findings and progressive cardiomyopathy comparable to the cardiac involvement of DMD patients [20-23]. In this respect, GRMD is a useful model to explore cardiac involvement, but GRMD is very difficult to maintain because of their severe phenotypes. Mild phenotypes can be expected in small sized dogs such as Beagle, indicated by the cross-bred study by Valentine *et al*. [20]. Moreover, medium-sized Beagle is easy to handle or raise than GRMD, therefore they have definite advantages in animal housing or welfare. Therefore, we established a Beagle-based dog colony named canine X-linked muscular dystrophy in Japan (CXMD_J_) [24]. In CXMD_J_, involvement of the temporalis and limb muscles is observed from 2 months of age, and macroglossia, dysphagia, drooling, and joint contracture are apparent from 4 months of age; the phenotypes of CXMD_J _are thus almost comparable to GRMD [25]. In this study, we investigated the cardiac phenotypes in CXMD_J _using electrocardiography, echocardiography, and pathological examinations. Abnormalities on echocardiogram and cardiac pathology were detected from 12 months of age; however, the distinct deep Q-waves in leads II, III, and aVF on ECG were consistently observed by 6–7 months of age in all CXMD_J _dogs examined. The cardiac phenotypes of CXMD_J _were identical to but milder than those of GRMD described in the literature. Thus, CXMD_J _may also be a suitable animal model for elucidation of the above-mentioned problems.

## Methods

### Animals

We imported frozen GRMD semen and artificially inseminated a Beagle bitch. The carriers produced were mated with unrelated Beagles, and a Beagle-based canine X-linked muscular dystrophy (CXMD_J_) breeding colony was established [24]. In this study, four normal male and eight affected male dogs of the third generation (G3) between 2 to 21 months of age were examined. All of the affected and normal dogs were descendents of a single affected male, and were part of the CXMD_J _breeding colony at the General Animal Research Facility, National Institute of Neuroscience, National Center of Neurology and Psychiatry (NCNP) (Tokyo, Japan) or the Chugai Research Institute for Medical Science, Inc. (Nagano, Japan). The clinical and histopathological characteristics, except for cardiac involvement, of CXMD_J _dogs were recently described [25]. These dogs were cared for and treated in accordance with the guidelines provided by the Ethics Committee for the Treatment of Laboratory Middle-Sized Animals of the National Institute of Neuroscience, NCNP (Tokyo, Japan) or the Ethics Committee for Treatment of Laboratory Animal of Chugai Pharmaceutical Co., Ltd. (Tokyo, Japan). These studies were also approved by the Ethics Committee for the Treatment of Laboratory Middle-Sized Animals of NCNP (approved No. 13-03, 14-03, 15-03, 16-03, 17-03, and 18-03). All experiments were performed with consideration for preventing unnecessary pain.

### Genotyping of CXMD_J _allele

Each affected or normal male dog was identified by genotyping. A snapback method of single-strand conformation polymorphism analysis was used to determine the GRMD allele as described previously [26].

### Measurement of serum creatine kinase (CK)

Blood samples were obtained from the cephalic vein at sacrifice. Serum CK level was measured by colorimetric assay using a FDC3500 clinical biochemistry analyzer (FujiFilm Medical Co., Tokyo, Japan).

### Electrocardiographic studies

Leads I, II, III, aVR, aVL and aVF were recorded in the right lateral recumbency using an ECG-922 electrocardiograph (Nihon Koden, Tokyo, Japan) [27]. All ECGs were obtained at a paper speed of 50 mm/sec and calibration of 10 mm/mV. First, the electrocardiography were performed in two CXMD_J _(III-302MA, III-303MA) and one normal littermate (III-301MN) dogs at 2, 3, 4, 6, 9, 12, 15, 18, and 21 months of age, and the heart rate (HR), intervals of PQ and QRS, and Q/R ratios were measured. However, in normal control and in CXMD_J_, Q waves were not prominent in leads aVR and aVL, therefore, we measured the Q/R ratios in leads I, II, III and aVF. Next, we compared the HR, intervals of PQ and QRS, or Q/R ratios in I, II, III and aVF in eight CXMD_J _and four normal dogs at 6–7 months of age.

### Echocardiographic studies

M-mode and two-dimensional echocardiography was performed using an EUB-8000 echocardiograph (Hitachi Medical Corporation, Tokyo, Japan). The thickness of the interventricular septum (IVS) and left ventricular posterior wall (LVPW) at end-diastole, left ventricular internal dimension at end-diastole (LVIDd) and systole (LVIDs), and fractional shortening (FS) were examined on normal and CXMD_J _dogs using M-mode echocardiography. We calculated the M-mode parameters based on multiple measurements of 5 consecutive heart cycles, or 3 or 5 representative heart cycles. We examined the parameters mentioned above and myocardial echogenicity in two CXMD_J _(III-302MA, III-303MA) and one normal littermate (III-301MN) dogs at 2, 3, 4, 6, 9, 12, 15, 18, and 21 months of age. We also examined the parameters mentioned above and myocardial echogenicity in six CXMD_J _(III-D53MA, III-D55MA, III-1803MA, III-D38MA, III-D02MA, III-D08MA) and three normal dogs (III-D56MN, III-1804MN, III-D03MN) at the time point just before euthanasia. Among those dogs, myocardial echogenicity in one CXMD_J _(III-D02MA) and its normal littermate (III-D03MN), and another CXMD_J _(III-D08MA) dogs were also examined at various time points.

### Macroscopic and histopathological analyses

All dogs in this study underwent cardiac histological analysis. After a dog was given an overdose of intravenous pentobarbital, the whole heart was removed and immediately fixed in 15% buffered formalin. Formalin-fixed hearts were cross-sectioned, and samples were taken from the right and left ventricles at the apical papillary muscle and basal levels (each level containing the interventricular septum, anterior wall, lateral wall, and posterior wall). The tissue blocks were embedded in paraffin, and 10 μm sections were stained with hematoxylin and eosin or Masson's trichrome stain. Photographs were taken with a DAS Mikroskop LEITZ DMRB microscope (Leica, Wetzlar, Germany), using a digital still camera system HC-2500 (FujiFilm, Tokyo, Japan).

### Statistics

Data are expressed as means +/- SE. Student's *t *test was used to evaluate differences between the two groups. A *p *value of less than 0.05 was considered to indicate statistical significance.

## Results

### Clinical profiles of CXMD_J_

We recently reported the detailed clinical and histopathological characteristics of CXMD_J _except for cardiac phenotypes [25]. None of the dogs in the present study showed clinical signs of heart failure, and no murmur was present on auscultation in any CXMD_J _dog examined. We evaluated body and heart weight, the ratio of heart to body weight, and serum CK in eight CXMD_J _and four normal male dogs aged 6–21 months just before euthanasia (Table 1). There were no differences in body and heart weight and heart/body weight ratio between normal and CXMD_J _dogs. Serum CK levels in the CXMD_J _dogs ranged from 12,500 to 13 8,000 IU/1. These values were significantly different from those in normal control dogs (60 to 515 IU/1). One 9-month-old CXMD_J _dog, III-D55MA, did not show any signs of respiratory or cardiac failure. When we tried to record a routine ECG of the dog, the dog struggled to escape from recording and then ceased moving. Immediately afterwards, we recorded ECG and the monitor showed an idioventricular rhythm. The dog died despite attempted cardiopulmonary resuscitation.

**Table 1:** Clinical profiles of normal and CXMD_J _male dogs

	Age (mo)	BW(g)	HW(g)	HW/BW (%)	Serum CK (IU/I)
Normal dogs					
III-D56MN	6	12.0	95.1	0.97	515
III-1804MN	7	13.6	110.0	0.81	215
III-D03MN	14	13.1	127.0	0.84	215
III -301 MN	21	14.4	120.0	0.96	60

CXMD_J _dogs					
III-D53MA	6	9.6	91.9	0.90	63,100
III-D55MA	7	10.0	80.0	0.80	42,000
III-1803MA	9	14.4	128.6	0.69	69,100
III-D38MA	12	11.4	78.8	1.01	138,000
III-D02MA	15	9.1	92.0	0.87	17,600
III-D08MA	15	12.0	104.3	0.97	40,700
III-302MA	21	12.4	120.0	0.86	12,500
III-303MA	21	13.9	120.0	0.80	23,000

BW, body weight; HW, heart weight; HW/BW, heart weight/body weight ratio; * *p *< 0.01 normal dogs *vs *CXMD_J _dogs

### Electrocardiographic findings

**Figure 1:**
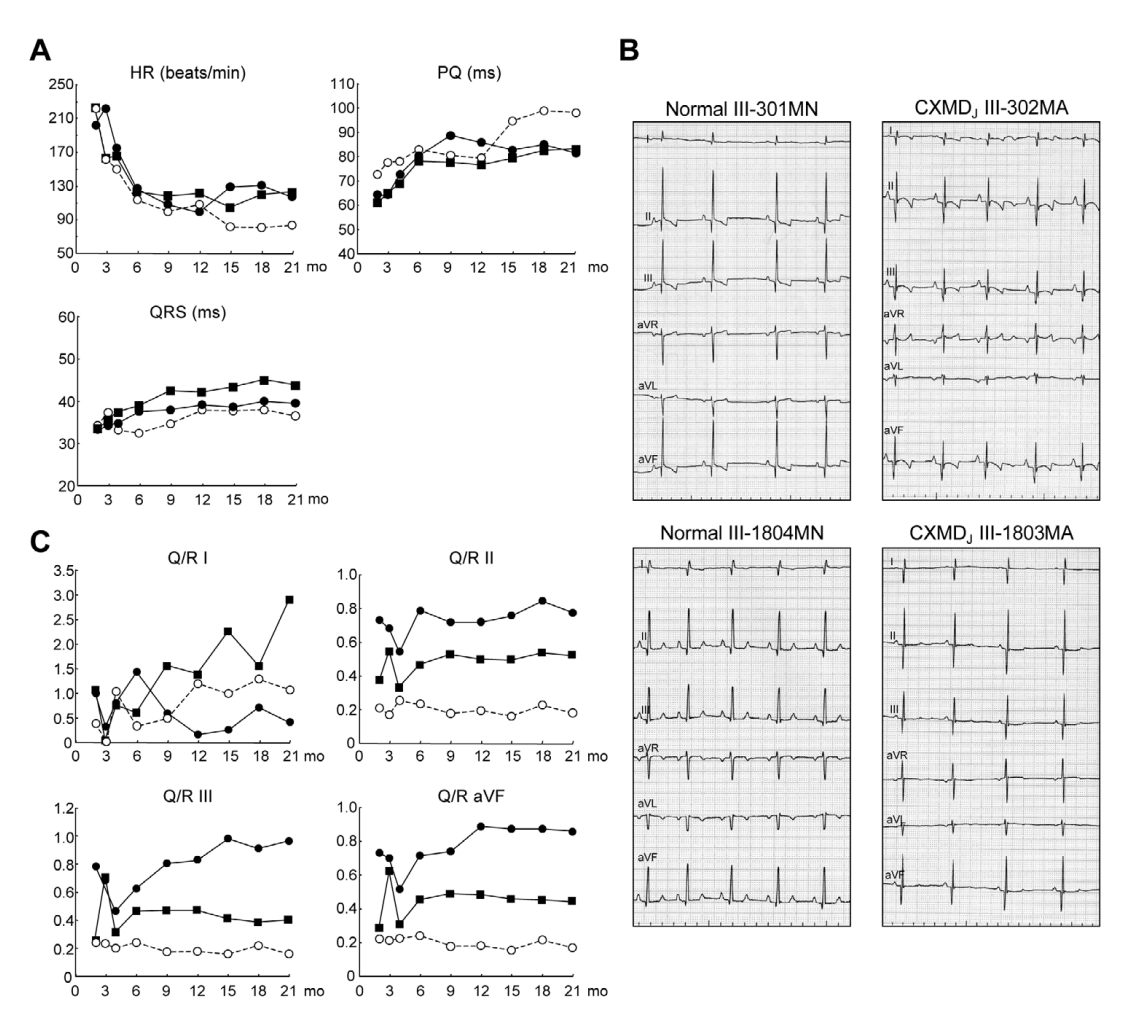
**Electrocardiographic findings in CXMD_J _A**: Sequential studies in electrocardiographic parameters with advancing age in normal and CXMD_J _dogs. Heart rate (HR) (beats/min), PQ interval (ms), and duration (ms) of QRS complex on ECG in a normal littermate III-301MN (open circle), and CXMD_J _dogs III-302MA (closed circle) and III-303MA (closed square) at 2, 3, 4, 6, 9, 12, 15, 18, and 21 months of age. **B**: Representative ECGs in normal and CXMD_J _male dogs. ECGs were recorded from normal dogs, III-301MN and III-1804MN, and CXMD_J _dogs, III-302MA and III-1803MA, at 6 months of age. Distinct deep Q waves were present in the CXMD_J _dogs. Leads were recorded at 50 mm/s, 1 cm/mV. **C**: Sequential studies in Q/R ratios with advancing age in limb leads I, II, III, and aVF in normal and CXMD_J _dogs. Q/R ratios in limb leads I, II, III, and aVF in a normal littermate III-301MN (open circle), and the CXMD_J _dogs III-302MA (closed circle) and III-303MA (closed square) at 2, 3, 4, 6, 9, 12, 15, 18, and 21 months of age.

**Figure 2:**
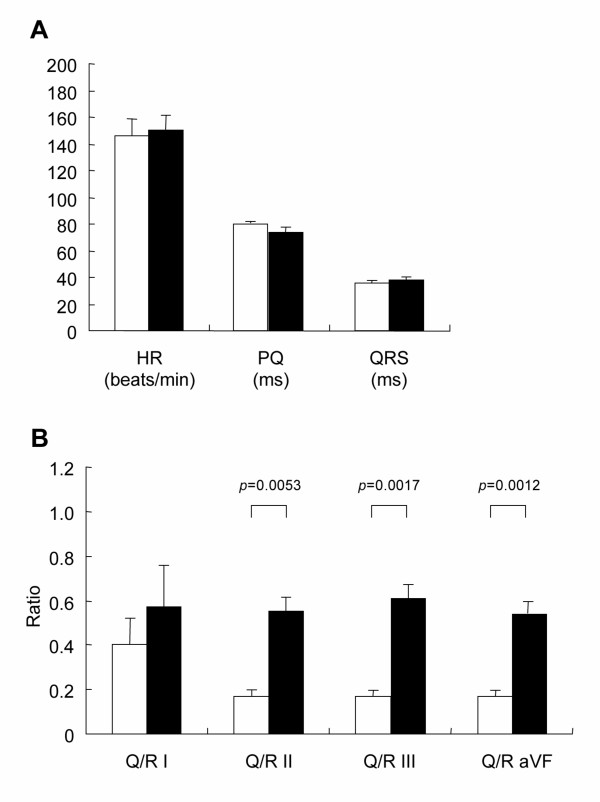
**Comparison of electrocardiographic parameters and Q/R ratios between normal and CXMD_J _dogs at 6–7 months of age A. **Heart rate (HR) (beats/min), PQ interval (ms), and duration of QRS complex (ms) on ECG in normal (n = 4) and CXMD_J _(n = 8) dogs at 6–7 months of age. White columns indicate normal dogs, and black columns represent CXMD_J _dogs. Bar shows mean +/- SE. **B. **Q/R ratios in limb leads I (Q/R I), II (Q/R II), III (Q/R III), and aVF (Q/R aVF) on ECG in normal (n = 4) and CXMD_J _(n = 8) dogs at 6–7 months of age. White columns indicate normal dogs, and black columns represent CXMD_J _dogs. Bar shows mean +/- SE.

The HR and PQ intervals of the affected littermates were no different from those recorded from a normal littermate at 12 months of age, but we detected an increase in HR and a decrease in PQ interval in the affected dogs after 15 months of age (Fig. 1A). The HR and PQ intervals were negatively correlated both in normal and affected dogs (data not shown). The QRS interval in the affected dogs did not differ from that in the normal littermate at any age (Fig. 1A). Prominent deep Q-waves were observed in limb leads II, III, and aVF in some CXMD_J _dogs, but not in the normal littermates, as shown in Fig. 1B. The Q/R ratios were definitely increased in the affected littermates at 6 months of age or older compared with the normal littermate (Fig. 1C). In all normal and CXMD_J _dogs at 6–7 months of age, the HR, and intervals of PQ and QRS were not different between the two groups of dogs (Fig. 2A), but the Q/R ratios in leads II, III, and aVF in the affected dogs were significantly higher than those in the normal dogs (Fig. 2B).

### Echocardiographic findings

**Figure 3:**
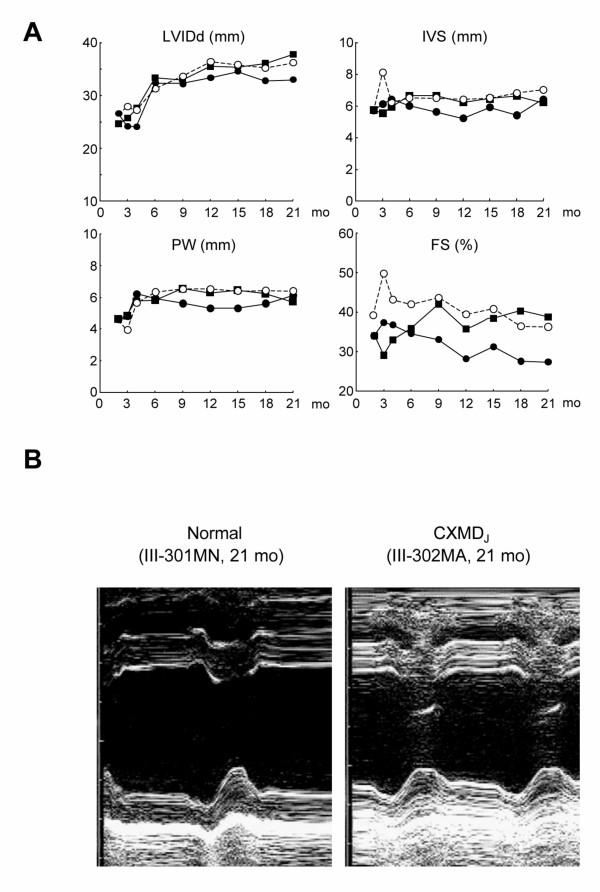
**Cardiac function by echocardiography in CXMD_J _A**: Sequential studies in echocardiographic parameters with advancing age in normal and CXMD_J _dogs. LVIDd (mm), IVS and PW thickness (mm), and FS (%) in a normal littermate III-301MN (open circle), and the CXMD_J _dogs III-302MA (closed circle) and III-303MA (closed square) at 2, 3, 4, 6, 9, 12, 15, 18, and 21 months of age. **B**: M-mode echocardiogram in a normal littermate III-301MN, compared to the CXMD_J _dog III-302MA at 21 months of age. Hypokinesis of the left ventricular posterior wall was observed in the CXMD_J _dog.

The thickness of LVIDd, IVS, and PW in two CXMD_J _(III-302MA, III-303MA) were not different from those in a normal littermate (III-301MN) by sequential analysis with advancing age (Fig. 3A). Those parameters were not different between other six CXMD_J _and three normal dogs, when examined just before euthanasia (Table 2). FS in III-302MA decreased with advancing age, and the value (27.3%) at 21 months of age was lower than that of the normal littermate, but was within normal range reported previously [28,29]. FS in the other seven CXMD_J _were normal, even just before euthanasia (Table 2). A mild hypokinesis of the left ventricular wall was detected in III-302MA at 21 months of age (Fig. 3B), but any clinical signs had not been developed in the dog.

**Table 2:** Echocardiographic findings in normal and CXMD_J _male dogs

	Age (mo)	LVIDd (mm)	LVIDs (mm)	IVS (mm)	PW (mm)	FS (%)
Normal male dogs						
III-D56MN	6	34.2	12.5	6.6	5.6	63.5
III-1804MN	7	30.7	18.9	8.2	7.4	38.4
III-D03MN	14	32.8	16.8	10.0	9.4	48.7
III-301 MN	21	36.1	23.0	7.0	6.4	36.2

CXMD_J _male dogs						
III-D53MA	6	29.7	19.7	8.0	7.2	33.8
III-D55MA	7	28.7	15.4	6.3	6.3	46.5
III-1803MA	7	32.5	16.7	5.8	7.5	48.5
III-D38MA	12	30.3	18.8	8.4	5.8	37.8
III-D02MA	15	27.5	17.3	9.1	8.8	37.0
III-D08MA	15	39.5	24.9	5.9	5.9	36.9
III-302MA	21	32.9	23.9	6.4	6.1	27.3
III-303MA	21	37.6	23.1	6.2	5.7	38.6

Age, age at examination; LVIDd, LV internal dimension diastolic; LVIDs, LV internal dimension systolic; IVS, intraventricular septum thickness; PW, posterior wall thickness; FS, fractional shortening

**Figure 4:**
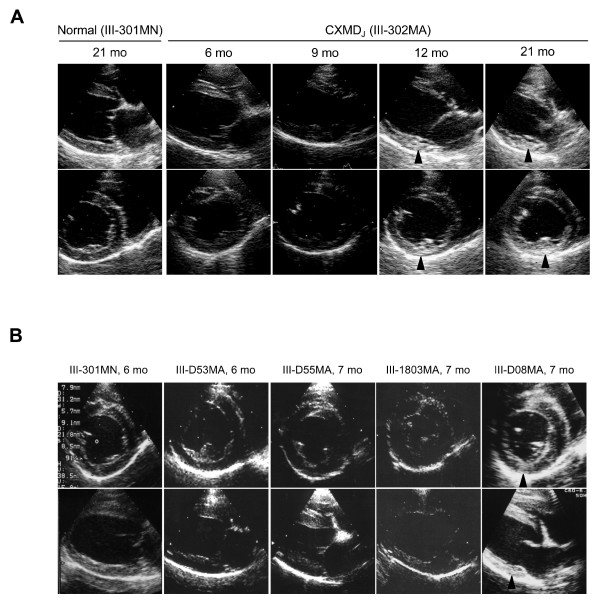
**Echogenicity in CXMD_J _A**: Sequential studies in echogenicity with advancing age by two-dimensional echocardiography in a normal dog III-301MN, and a CXMD_J _dog III-302MA, at 6–21 months of age. Hyperechoic lesions (arrowheads) of the left ventricular posterior wall were detected in the CXMD_J _dog at 12 months of age and older.**B**: Two-dimensional echocardiograms of a normal dog III-301MN at 6 months of age, and four CXMD_J _dogs III-D53MA, III-D55MA, III-1803MA, and III-D08MA at 6 to 7 months of age. The hyperechoic lesion (arrowhead) was detected only in the left ventricular posterior wall of III-D08MA.

The sequential studies of myocardial echogenicity with advancing age in III-302MA and in III-303MA demonstrated that the hyperechoic lesions in the left ventricular posterior wall were seen at 12 months of age or older (Fig. 4A, Table 3). In the subsequent examinations of six CXMD_J_, we found the hyperechoic lesion in a CXMD_J_, III-D08MA, at 5 months of age (Fig. 4B, Table 3), however the hyperechoic lesion was not detected in other four CXMD_J _at 5 to 7 months old (Fig. 4B, Table 3). One CXMD_J_, III-D38MA, did not reveal any hyperechoic lesions when examined at 12 months of age, but has not been examined at 5 to 7 months of age (Table 3). Taken these echocardiographic data, it is considered that the cardiac functions in CXMD_J _were basically maintained well by 21 months of age, despite showing hyperechoic lesions of the left ventricle in limited numbers of CXMD_J_.

**Table 3:** Echogenicity of left ventricular posterior wall in normal and CXMD_J _male dogs

	Months of age (mo)																			
	
	2	3	4	5	6	7	8	9	10	11	12	13	14	15	16	17	18	19	20	21
Normal dogs																				
III-D56MN					(-)*															
III-1804MN					(-)	(-)*														
III-D03MN	(-)			(-)		(-)			(-)			(-)	(-)*							
III-301 MN	(-)	(-)	(-)		(-)			(-)			(-)			(-)			(-)			(-)*

CXMD_J _dogs																				
III-D53MA					(-)*															
III-D55MA						(-)		*												
III-1803MA					(-)	(-)*														
III-D38MA											(-)*									
III-D02MA	(-)			(-)		(-)					(-)	(-)		(-)*						
III-D08MA	(-)			(+)		(+)					(+)	(+)		(+)*						
III-302MA	(-)	(-)	(-)		(-)			(-)			(+)			(+)			(+)			(+)*
III-303MA	(-)	(-)	(-)		(-)			(-)			(+)			(+)			(+)			(+)*

Hyperechoic lesion +, positive; -, negative; Asterisk in each CXMD_J _dog shows age at euthanasia.

### Macroscopic and histopathological findings

**Figure 5:**
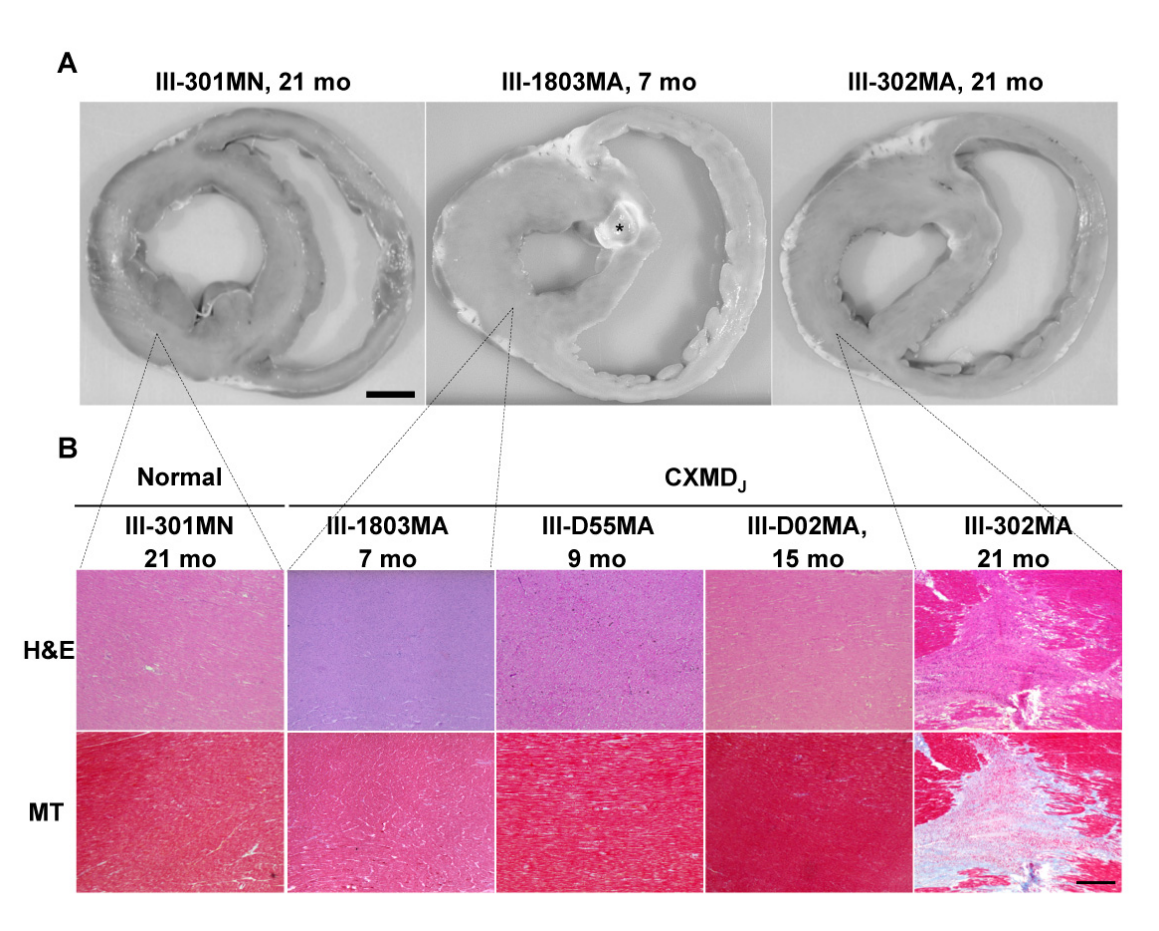
**Macroscopic and histopathological findings in CXMD_J _hearts A. **Macroscopic examinations of the base of the formalin-fixed hearts of a normal littermate III-301MN at 21 months and CXMD_J _dogs, III-1803MA at 7 months and III-302MA at 21 months of age. *Aortic valve. Bar shows 1 cm. **B. **Hematoxylin and eosin (H&E) and Masson's trichrome (MT) staining for histopathological evaluation of the left ventricular posterior wall in a normal littermate, III-301MN at 21 months and the CXMD_J _dogs, III-1803MA at 7 months, III-D55MA at 9 months, III-D02MA at 15 months, and III-302MA at 21 months of age. Posterior walls of left ventricles of both III-D55MA and III-D02MA were macroscopically normal (data not shown). Bar shows 200 μm.

The right and left ventricular walls were examined macroscopically and histopathologically in four normal and eight affected male dogs at the ages shown in Table 1. The base view of the formalin-fixed heart did not show any macroscopic lesions in III-1803MA at 7 months and III-302MA at 21 months of age (Fig. 5A) like other affected dogs (data not shown). No histopathological abnormality was found in the posterior wall of the left ventricle of affected dogs III-1803MA, III-D55MA, and III-D02MA (Fig. 5B), and other affected dogs under the age of 12 months (III-D53MA, III-D38MA). On the other hand, moderate fibrosis in the left ventricular wall, especially on the posterior side, was detected in an affected dog, III-302MA, at 21 months (Fig. 5B) as well as in III-D08MA at 15 months and III-303MA at 21 months of age (data not shown). We found that the right ventricular walls were kept intact in all CXMD_J _dogs examined.

## Discussion

In electrocardiographic findings, an increased HR and a shortened PQ interval have been reported in both DMD patients [13] and GRMD [22]. These findings were also observed in CXMD_J _dogs. Increased sympathetic activity and decreased parasympathetic activity have been observed in DMD patients and are associated with disease progression [30]; therefore, autonomic dysfunction in dystrophin deficiency might affect these parameters. It has been reported that HR is negatively correlated with PQ interval in normal Beagle dogs and it may be ascribed to a parasympathetic input at the level of the AV node [31]. The negative correlation between HR and PQ intervals was also found in affected dogs, indicating the parasympathetic input was maintained well even in affected dogs at AV node level. The QRS duration was within normal limits in the CXMD_J _dogs, which is compatible with most cases of DMD [13]. Another peculiar electrocardiographic finding in DMD is the deep and narrow Q-waves in I, aVL and V6 or in II, III and aVF [10,13,16,32]. CXMD_J _dogs also showed prominent Q-waves and increases in the Q/R ratio in leads II, III, and aVF, findings that are consistent with those in GRMD [23]. In all CXMD_J _dogs examined, the distinct deep Q-waves were recognized by 6–7 months of age, which is earlier than the other abnormal electrocardiographic parameters, and the Q/R ratio in affected dogs remained high from 6 to 21 months of age. Actually, the prominent Q-wave and increase in Q/R ratio were also detected in some of the CXMD_J _dogs at around 2 months of age (Fig. 1C), but it is difficult to evaluate the degree of the Q/R ratio increase before 3 months of age because the QRS vector is almost exclusively directed to the right and varies significantly in the weeks after birth [33]. A previous report described GRMD dogs ranging from 6 months to > 2 years as having deep Q-waves and increased Q/R ratios in leads II, III, and aVF [23]. The Q-waves, however, might have been seen earlier and regarded as normal variants or not have been considered important for the reasons mentioned above.

Hyperechoic lesions indicating myocardial fibrosis in the posterobasal left ventricular wall have been detected by echocardiography in GRMD dogs as well as DMD patients [22,23]. Moise *et al*. reported that hyperechoic lesions were first detected in eight of eleven GRMD dogs (73%) by 6–7 months of age and that they correlated with histologically recognizable areas of mineralization and corresponded to the progression of fibrosis [23]. In our study, one of eight of CXMD_J _dogs showed a hyperechoic lesion in the left ventricular posterior wall, but the rest had not by the age of 6–7 months (Table 3, Fig. 4). The hyperechoic lesion in the left ventricular posterior walls was detected in both III-302MA and III-303MA, but not early as 12 months of age (Table 3). The results of echocardiography indicated that the cardiac involvement in CXMD_J _is milder than that in GRMD. Echocardiography did not reveal particular left ventricular dysfunction in any CXMD_J _dog by 21 months of age, but a mild hypokinesis of the left ventricular wall was observed in III-302MA at 21 months of age (Fig. 3B). The dysfunction found in the dog, however, was mild and the dog had no cardiac symptom. Moise *et al*. reported that three of the six GRMD dogs > 2 years of age showed a decrease in fractional shortening, but did not mention at what age the abnormal cardiac findings appeared.

Previous studies of morphology in GRMD showed that myocardial involvement is initially found in the left posterobasal ventricular wall, similar to that of patients with DMD [21-23]. Valentine *et al*. reported that GRMD dogs at 6.5 months of age had acute severe lesions with focal myocardial mineralization associated macrophages and giant cells in the left ventricular papillary muscle and left ventricular wall [22]. Moreover, GRMD dogs at 12 months of age or older demonstrated prominent myocardial fibrosis in more widespread lesions [22]. The myocardial fibrosis of the left ventricular wall in the older stage of CXMD_J _dogs was consistent with that in DMD patients and GRMD dogs. The change was detected at 15 months of age or older in the CXMD_J _(III-D08MA, III-302MA, and III-303MA), although III-D08MA showed a hyperechoic lesion at 5 months of age or older (Table 3). The cardiac involvement in CXMD_J_, therefore, was milder and slowly progressed than that in GRMD, although a longer period evaluation of large numbers of CXMD_J _will be needed to conclude the mild cardiac phenotypes of CXMD_J_.

Why is the cardiac involvement in CXMD_J _milder than that in GRMD? Valentine *et al*. reported that skeletal muscle involvement in small dystrophic dogs was milder than that in large ones [19]. Several reports on dystrophic features have hypothesized that the clinical severity may be associated with growth rate [34] or muscle fiber diameter [35]. Living in a cage rather than running free could also affect the cardiac phenotypes of CXMD_J _because physical exercise promotes cardiac involvement in dystrophin-deficient *mdx *mice [36]. The difference in the genetic background between GRMD, golden retriever and CXMD_J_, Beagle might also affect the disease progression.

**Figure 6:**
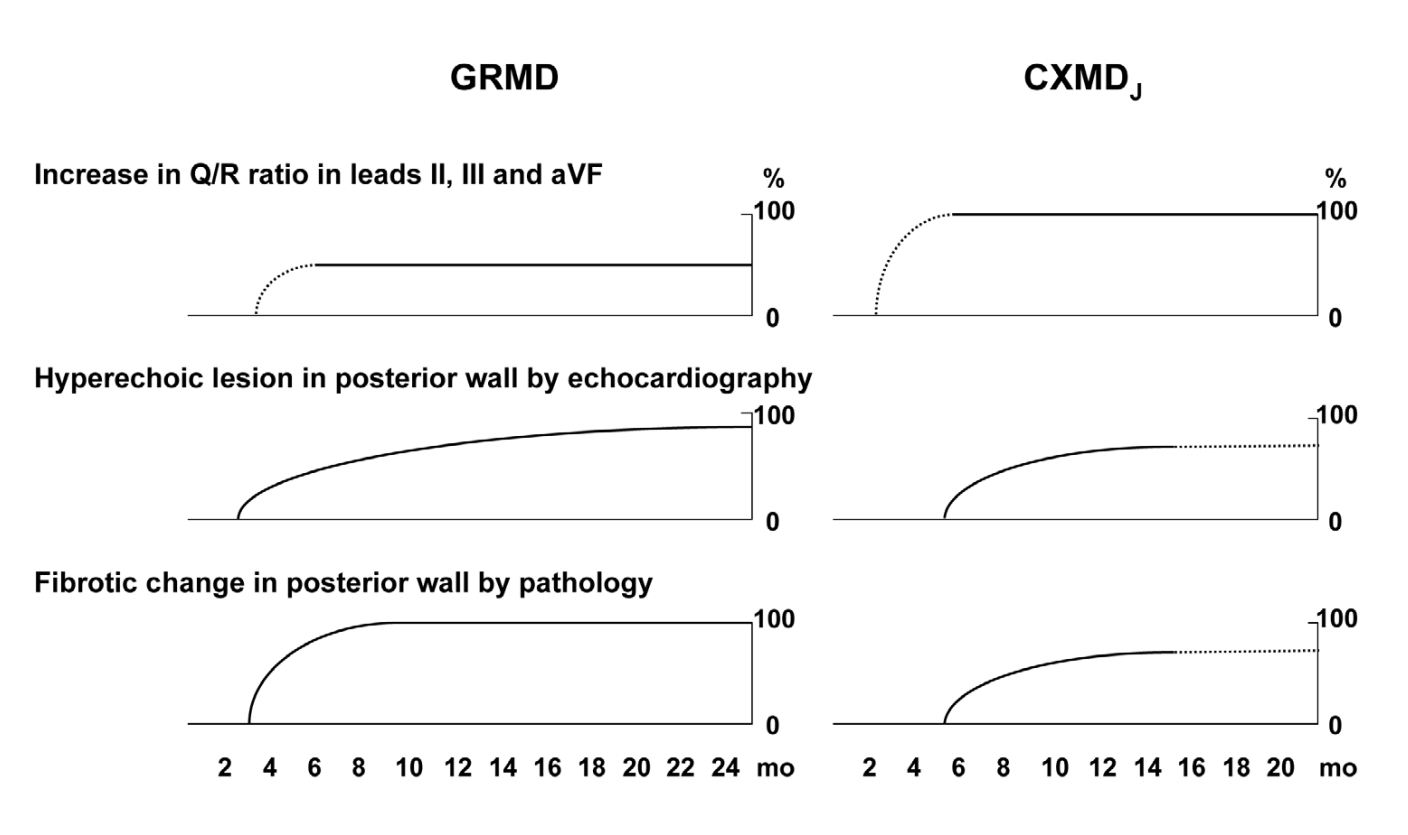
**Comparison of cardiac involvement between GRMD and CXMD_J _with advancing age**. Subjects were compared as follows: increase in Q/R ratio in leads II, III, and aVF in ECG, hyperechoic lesion in posterior wall by echocardiography, and fibrotic change in left ventricular posterior wall by pathology. The data on GRMD was based on the previous literature [22–24]. It is difficult to evaluate Q/R ratio in early stage of GRMD and CXMD_J_. It is also difficult to evaluate hyperechoic lesion in echocardiogram and fibrotic change in pathology at late stage of CXMD_J _due to small numbers of examination (n < 3).

The prominent deep Q-waves seen in both DMD and GRMD have been attributed to a reduction in or a loss of electromotive force caused by scarring of the posterobasal region of the left ventricle [8,9,17]. In our study, the deep Q-waves and increases in the Q/R ratio in CXMD_J _preceded the lesions seen on echocardiogram and histopathology, as shown in Fig. 6. Considering this result, the origin of the distinctive Q-waves might not be associated with the myocardial lesion in the posterobasal left ventricular wall. It has recently been reported that expression of a transgene in *mdx *mice for neuronal nitric oxide synthase (nNOS), which occurs as a secondary loss in dystrophin deficiency, decreased cardiac inflammation and fibrosis resulting in amelioration of both cardiac function and electrocardiographic abnormalities, including deep Q-waves [37]. Perloff *et al*. suggested that the alteration of a particular ionic current by lack of specific membrane proteins associated with dystrophin might participate in electrocardiographic changes [17]. We will not therefore deny that minimal myocardial damage could be associated with the pathogenesis of deep Q-waves, but our results suggest that an investigation of the conduction and cardiovascular systems will also be needed to explore the pathophysiology of the deep Q-waves in dystrophin-deficient heart. In this regard, CXMD_J _will be very useful to elucidate aspects of the dystrophin-deficient heart, but we may recognize that a longer period of time would be required to complete cardiac phenotypes in CXMD_J_.

## Conclusion

We demonstrated that the cardiac phenotypes of CXMD_J _are comparable to but milder than those of GRMD. Furthermore, we found for the first time that the distinct deep Q-waves precede detection of the left ventricular posterobasal lesion by echocardiography or histopathology. CXMD_J _may provide not only new insights into the mechanisms causing the abnormal Q-waves but also more information on the pathogenesis in the dystrophin-deficient heart.

## Competing interests

The author(s) declare that they have no competing interests.

## Authors' contributions

NY and NU carried out the electrocardiographic, echocardiographic, and pathological examination and drafted the manuscript. YF performed the electrocardiographic study. MY, KY and MRW participated in the necropsy and pathological examination. MN, YS, MT and AT participated in the maintenance of the dog colony and the design of the study. NM performed the pathological examination. YW participated in the design of the study. AN participated in the statistical analysis and drafted manuscript. ST participated in the design, planning and coordination of the study. All authors read and approved the final manuscript.

## Pre-publication history

The pre-publication history for this paper can be accessed here:


